# Characterization of a novel wood mouse virus related to murid herpesvirus 4

**DOI:** 10.1099/vir.0.017327-0

**Published:** 2010-04

**Authors:** David J. Hughes, Anja Kipar, Steven G. Milligan, Charles Cunningham, Mandy Sanders, Michael A. Quail, Marie-Adele Rajandream, Stacey Efstathiou, Rory J. Bowden, Claude Chastel, Malcolm Bennett, Jeffery T. Sample, Bart Barrell, Andrew J. Davison, James P. Stewart

**Affiliations:** 1 School of Infection and Host Defence, University of Liverpool, Liverpool L69 3GA, UK; 2 Department of Veterinary Pathology, University of Liverpool, Liverpool, L69 7ZJ, UK; 3 MRC Virology Unit, Institute of Virology, University of Glasgow, Church Street, Glasgow G11 5JR, UK; 4 The Wellcome Trust Sanger Institute, The Wellcome Trust Genome Campus, Cambridge CB10 1SA, UK; 5 Division of Virology, Department of Pathology, University of Cambridge, Tennis Court Road, Cambridge CB2 1QP, UK; 6 Laboratoire de Virologie, Faculté de Médecine, 29285 Brest, France; 7 Department of Microbiology and Immunology, The Pennsylvania State University College of Medicine, Hershey, PA 17033, USA

## Abstract

Two novel gammaherpesviruses were isolated, one from a field vole (*Microtus agrestis*) and the other from wood mice (*Apodemus sylvaticus*). The genome of the latter, designated wood mouse herpesvirus (WMHV), was completely sequenced. WMHV had the same genome structure and predicted gene content as murid herpesvirus 4 (MuHV4; murine gammaherpesvirus 68). Overall nucleotide sequence identity between WMHV and MuHV4 was 85 % and most of the 10 kb region at the left end of the unique region was particularly highly conserved, especially the viral tRNA-like sequences and the coding regions of genes *M1* and *M4*. The partial sequence (71 913 bp) of another gammaherpesvirus, Brest herpesvirus (BRHV), which was isolated ostensibly from a white-toothed shrew (*Crocidura russula*), was also determined. The BRHV sequence was 99.2 % identical to the corresponding portion of the WMHV genome. Thus, WMHV and BRHV appeared to be strains of a new virus species. Biological characterization of WMHV indicated that it grew with similar kinetics to MuHV4 in cell culture. The pathogenesis of WMHV in wood mice was also extremely similar to that of MuHV4, except for the absence of inducible bronchus-associated lymphoid tissue at day 14 post-infection and a higher load of latently infected cells at 21 days post-infection.

## INTRODUCTION

The most extensively characterized members of the family *Herpesviridae* that have hosts in the family Muridae are mouse cytomegalovirus (MCMV) and rat cytomegalovirus, which are classified in the genus *Muromegalovirus* of the subfamily *Betaherpesvirinae*, and murid herpesvirus 4 (MuHV4; also known as murine gammaherpesvirus 68, often abbreviated to MHV-68 or *γ*HV-68; species *Murid herpesvirus 4*), classified in the genus *Rhadinovirus* of the subfamily *Gammaherpesvirinae* (Davison *et al.*, 2009; Efstathiou *et al.*, 1990). Species in the genus *Rhadinovirus* also represent five herpesviruses of primates and one of ungulates. However, these viruses are not closely related to MuHV4, and the best estimate is that the lineages within the genus diverged approximately 60 million years ago (McGeoch *et al.*, 2005). At least three other murid herpesviruses have been reported, although these are unclassified at present (Davison *et al.*, 2009).

MuHV4 was originally isolated from bank voles (*Myodes glareolus*) and yellow-necked field mice (*Apodemus flavicollis*) in Slovakia (Blaskovic *et al.*, 1980; reviewed by Nash *et al.*, 2001). An epidemiological survey of MuHV4 infection in free-living rodents in the UK (Blasdell *et al.*, 2003) showed that MuHV4 is endemic in wood mice (*Apodemus sylvaticus*), but not bank voles, indicating that the wood mouse is a major natural host for this virus. Recent definitive molecular data has also shown that MuHV4 is present in free-living yellow-necked field and wood mice (Ehlers *et al.*, 2007).

In consideration of these observations, a wood mouse infection model was developed as an alternative to a model utilizing the laboratory (house) mouse (*Mus musculus*), which has been used to date for MuHV4 studies (Hughes *et al.*, 2010). In comparison with the BALB/c laboratory mouse, the features of MuHV4 infection in the wood mouse are: (i) after intranasal inoculation, viral titres achieved in the lung are approximately 1000-fold lower; (ii) replication is restricted to scattered alveolar epithelial cells and macrophages within focal granulomatous infiltrations, rather than being evident as a diffuse, T-cell-dominated, interstitial pneumonitis; (iii) latently infected lymphocytes are abundant in inducible bronchus-associated lymphoid tissue (iBALT); (iv) the spleens of wood mice show reduced splenomegaly and leukocytosis; (v) well-delineated secondary follicles with classical germinal centres are formed; and (vi) titres of neutralizing antibody to MuHV4 are significantly higher.

The present study focused on the isolation and genetic and biological characterization of a novel MuHV4-like virus. Two independent strains were examined, one isolated in the present study from wood mice in Cheshire, UK, and the other from a white-toothed shrew (*Crocidura russula*) in Brest, France (Chastel *et al.*, 1994).

## RESULTS

### Genomic characterization of wood mouse herpesvirus (WMHV) and Brest herpesvirus (BRHV)

Three distinct viruses were obtained from free-living murids captured in Cheshire, UK. The isolation and transmission electron microscopy (TEM) results are summarized in Table 1.

The TEM-positive samples gave rise to PCR products from the DNA polymerase (DPOL) gene, whilst the TEM-negative samples did not (data not shown). The sequences of the 213 bp amplicons originating from WM1, WM2, WM7 and WM8 DPOL were identical to each other, regardless of the tissue from which the viruses were isolated. These sequences (minus the primers: 160 bp) exhibited 89 % nucleic acid identity and 94 % predicted amino acid sequence identity to the corresponding region of MuHV4 DPOL. The FV1 DPOL amplicon was also 213 bp in size, and the 160 bp sequence (minus primers) was more closely related to MuHV4 than to any other herpesvirus, with 61 % nucleic acid and 54 % amino acid sequence identity. The HM4 virus DNA yielded a DPOL PCR product of 231 bp (178 bp minus primers) that was closely related to MCMV (strain Smith) DPOL, with 99 % nucleic acid and 100 % amino acid sequence identity. These results confirmed the identification of two novel gammaherpesviruses. The WM isolates were designated WMHV and the FV1 isolate was designated field vole herpesvirus (FVHV).

**Fig. 1.**
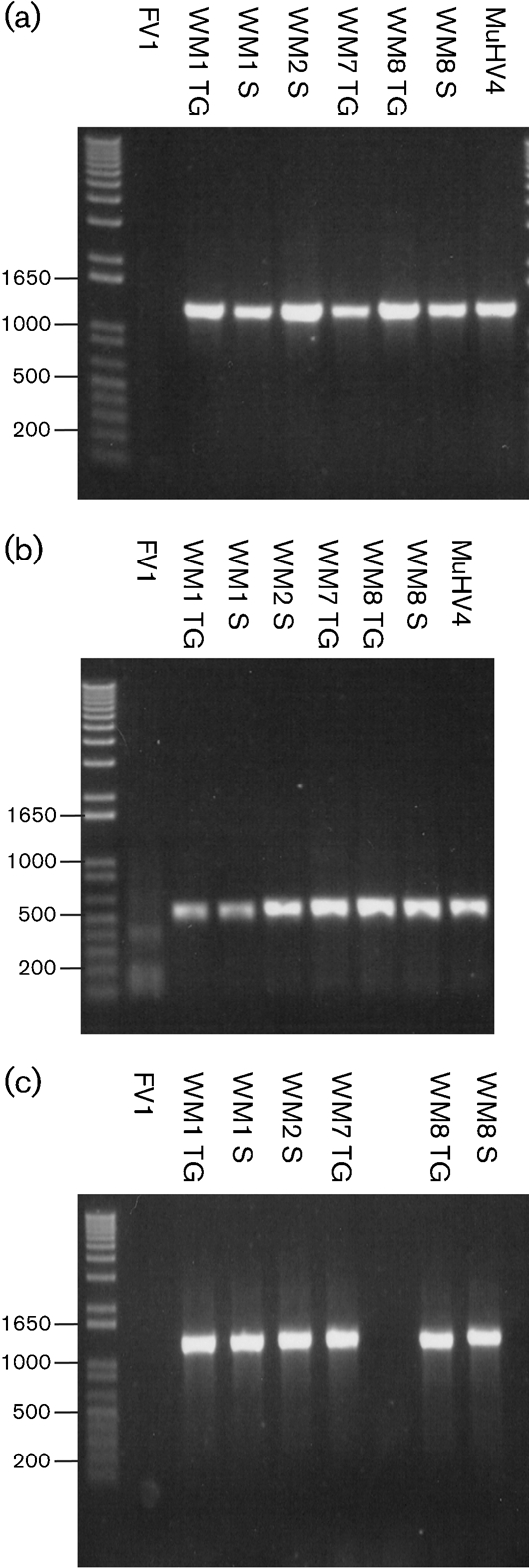
PCR amplification of the coding regions of the genes *M1* (a), *M2* (b) and *M3* (c) from viruses isolated from FV1, WM1, WM2, WM7 and WM8, in comparison with MuHV4. TG, Trigeminal ganglia; S, spleen.

An initial analysis of the coding regions of genes *M1*, *M2* and *M3*, which had been identified hitherto only in MuHV4, showed that cognate PCR products were detected in all WMHV isolates using MuHV4-specific primers, thus confirming the close relatedness of these isolates to MuHV4 (Fig. 1). In contrast, none of these genes was amplified from FVHV, consistent with its more distant relationship to MuHV4. The DNA sequences obtained from these PCR products for WMHV *M1* and *M3* were 97 and 94 % identical to MuHV4 *M1* and *M3*, respectively, whilst WMHV and MuHV4 *M2* sequences were more divergent, sharing only 83 % identity.

The complete nucleotide sequence of the WMHV genome was then determined. The genome structure deduced from the WMHV genome sequence was the same as that of MuHV4, consisting of a unique region (U) flanked at both ends by multiple direct repeats of a terminal repeat (TR). In WMHV and MuHV4, the size of U was 118 864 and 118 211 bp, respectively, and that of TR was 1244 and 1240 bp. Overall nucleotide sequence identity was 85 %. The predicted gene content of WMHV was the same as that for MuHV4, as represented by the most up-to-date annotation (GenBank accession no. NC_001826). A 71 913 bp segment of the BRHV genome was sequenced. This represented TR (1265 bp, plus a partial copy) linked to the left portion of U (70 439 bp) terminating within *ORF53*. The BRHV sequence was 86 and 99.2 % identical to the corresponding regions of the MuHV4 and WMHV genomes, respectively. The information used to annotate the genome sequences is shown in Supplementary Table S1 (available in JGV Online).

**Fig. 2.**
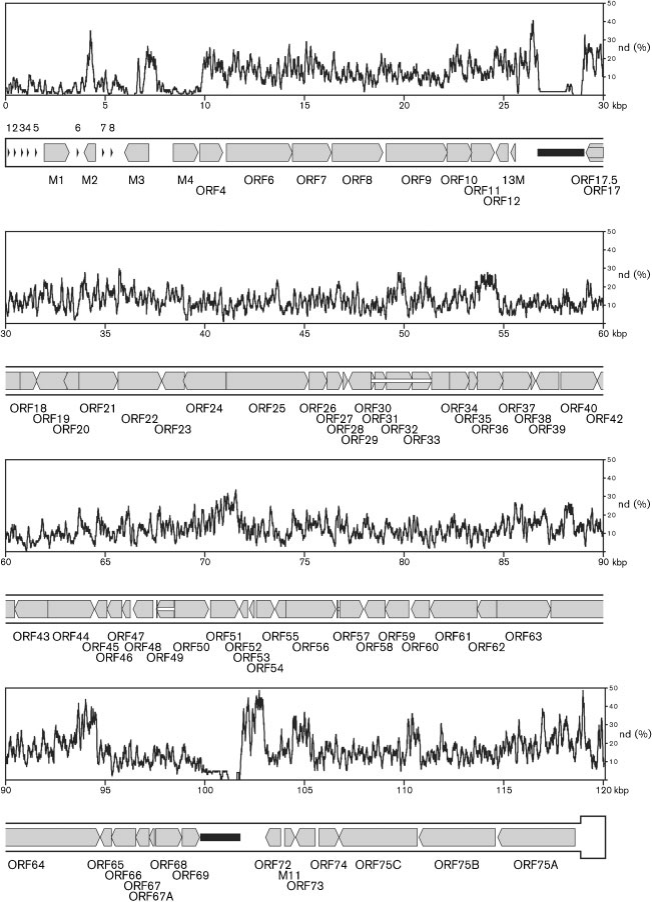
Variation between the genome sequences of WMHV and MuHV4. The lower part of the panels represents the genome, commencing in the first panel at the start of U and ending in the last panel with one copy of TR, which is shown in a thicker format. Protein-coding regions are depicted by shaded arrows, with connecting introns indicated by white horizontal bars and genes encoding the tRNA-like genes (1–8) shown as arrowheads. Internal tandem repeats are represented by black horizontal bars. The upper part of each panel shows the nucleotide divergence (nd) calculated for a 100 nt window, shifted by increments of 3 nt. A nucleotide position was counted as divergent if it differed between the two sequences; insertions and deletions were not scored.

**Fig. 3.**
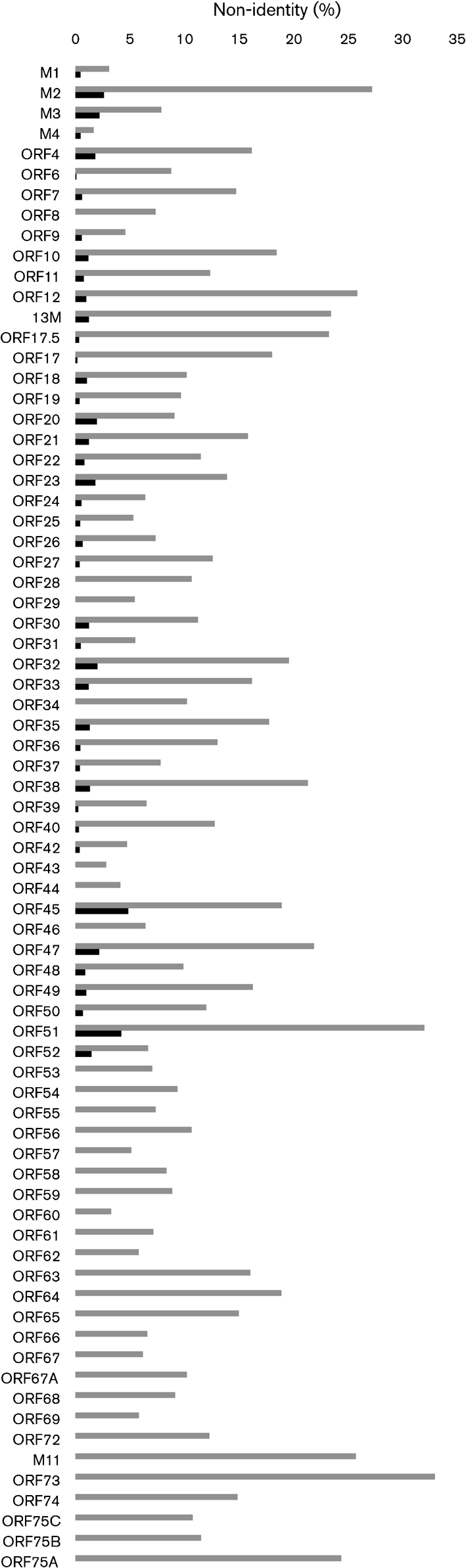
Divergence between the amino acid sequences of predicted protein-coding regions in WMHV, BRHV and MuHV4. The histogram illustrates sequence divergence (% non-identity) between the amino acid sequence of predicted protein-coding regions in WMHV and MuHV4 (grey bars, all coding regions) and WMHV and BRHV (black bars, coding regions up to *ORF52*).

**Fig. 4.**
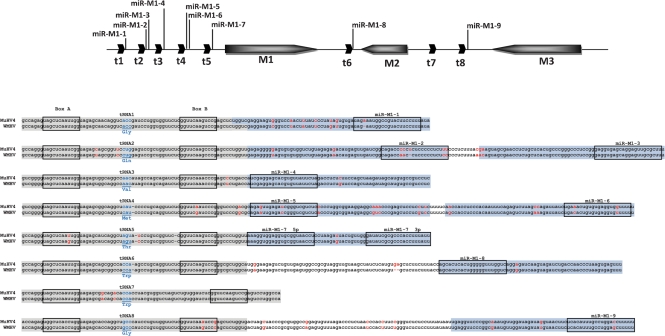
Alignment of the predicted nucleotide sequences of the tRNA-like genes and miRNAs from MuHV4 and WMHV. A diagrammatic representation of the genomic region showing the relative positions of these non-coding RNAs is shown at the top. The positions of the *M1–M3* ORFs and viral tRNA-like transcripts (t1–t8) are indicated by arrows. The positions of the miRNAs (miR-M1-1 to miR-M1-9) derived from primary transcripts of the tRNA-like RNAs are shown by vertical lines. Sequence alignments of the tRNA/miRNA transcripts are shown below. The sections processed to generate the tRNA-like molecules are shaded grey and pre-miRNAs are shaded blue. The positions of the A and B boxes of the RNA polymerase III promoters are shown by boxes, as are the positions of the processed miRNAs. The positions of the anti-codons in the tRNAs are shown in blue type. Differences between MuHV4 and WMHV are shown in red type. Data for MuHV4 are from Bowden *et al.* (1997) and Pfeffer *et al.* (2004).

**Fig. 5.**
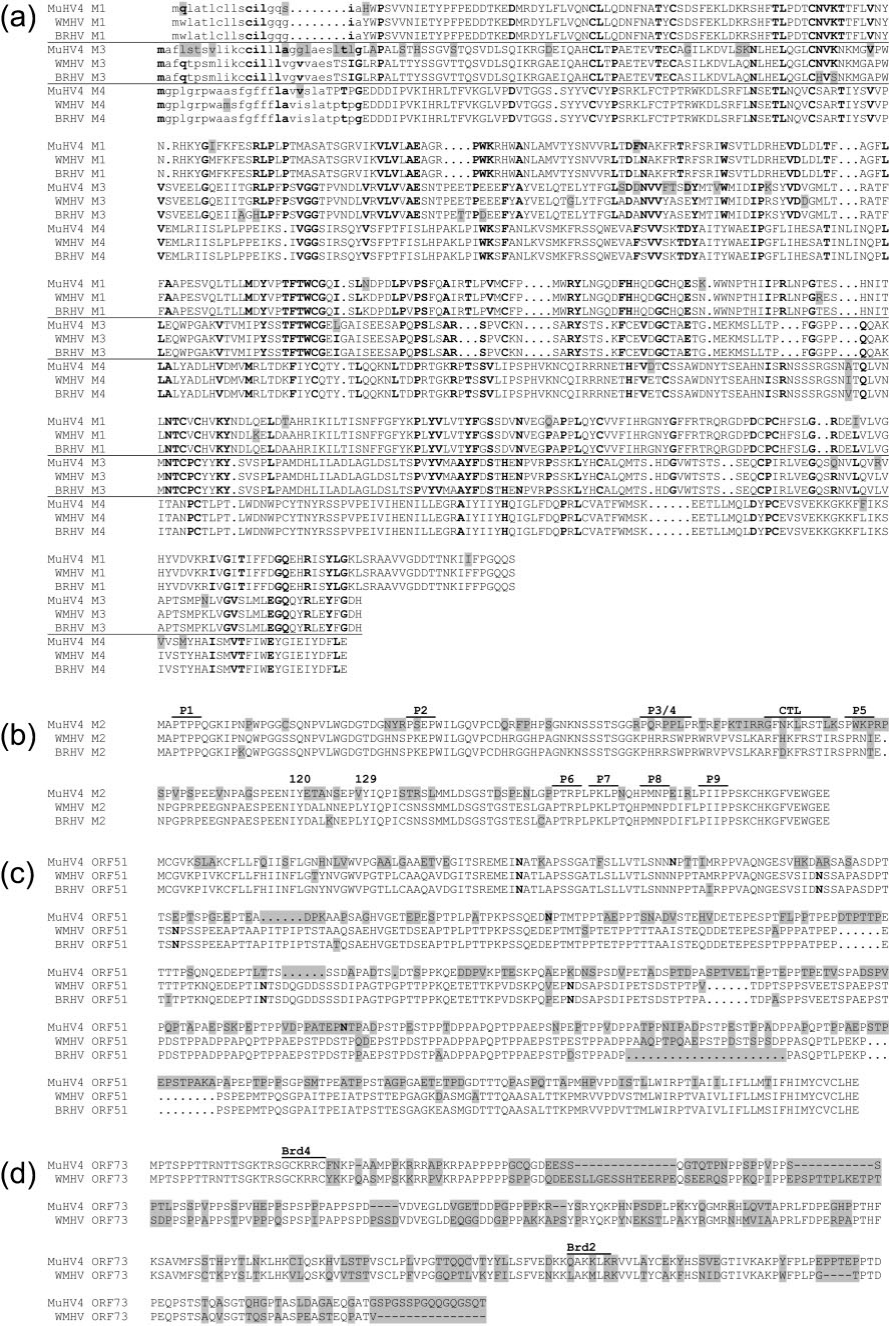
Alignments of the predicted amino acid sequences of M1, M3 and M4 (a), M2 (b), ORF51 (c) and ORF73 (d). Each individual alignment consists of the sequences from MuHV4, WMHV and BRHV, with residues that differ from the consensus (or from each other in the case of ORF73) shaded grey. In (a), the alignments for M1, M3 and M4 are aligned with each other because these three proteins are related via the residues in bold type; each sequence contains a predicted signal peptide (lower case). In (b), the positions of PXXP motifs (P1–P9), tyrosine residues 120 and 129 and the CD8 CTL epitope (CTL) are indicated above the sequence. In (c), the bold residues indicate potential *N*-linked glycosylation sites in gp150 encoded by *ORF51*. In (d), the positions of the Brd4- and Brd2-interacting domains of the ORF73 protein are shown above the sequences.

Fig. 2 shows a representation of DNA sequence identity along the entire WMHV and MuHV4 genomes, and Fig. 3 provides details on amino acid sequence identity between WMHV, BRHV and MuHV4 protein-coding regions. The most highly conserved regions between WMHV and MuHV4 included two sets of internal tandem repeats and the region from the left end of U to the end of the *M4* coding region, which included the eight viral tRNA-like genes (vtRNAs) (Bowden *et al.*, 1997). Although the vtRNAs were well conserved, there were functionally relevant differences in the sequences of microRNAs (miRNAs) 1, 2, 5, 6 and 9 that were derived from vtRNA primary transcripts (Fig. 4) (Pfeffer *et al.*, 2004). The most highly conserved coding regions at the amino acid sequence level were M4 (98.3 %), ORF43 (97.2 %), M1 (96.9 %) and ORF60 (96.7 %), and the least conserved were ORF73 (67.0 %), ORF51 (68.0 %) and M2 (72.8 %). In the comparable sequences, the most highly conserved coding regions at the amino acid sequence level between WMHV and BRHV were ORF8, ORF28, ORF29, ORF34, ORF43, ORF44 and ORF46 (each 100 %), and the least conserved were ORF45 (95.2 %), ORF51 (95.8 %) and M2 (97.4 %). Fig. 5 shows the amino acid sequence alignments for M1, M3 and M4 (which are related), and also M2, ORF51 and ORF73.

### Biological characterization of WMHV

The relative rates of growth of MuHV4 and WMHV in NIH3T3 cells were compared by determining a one-step growth curve as described previously (Macrae *et al.*, 2001) and were not significantly different (data not shown).

**Fig. 6.**
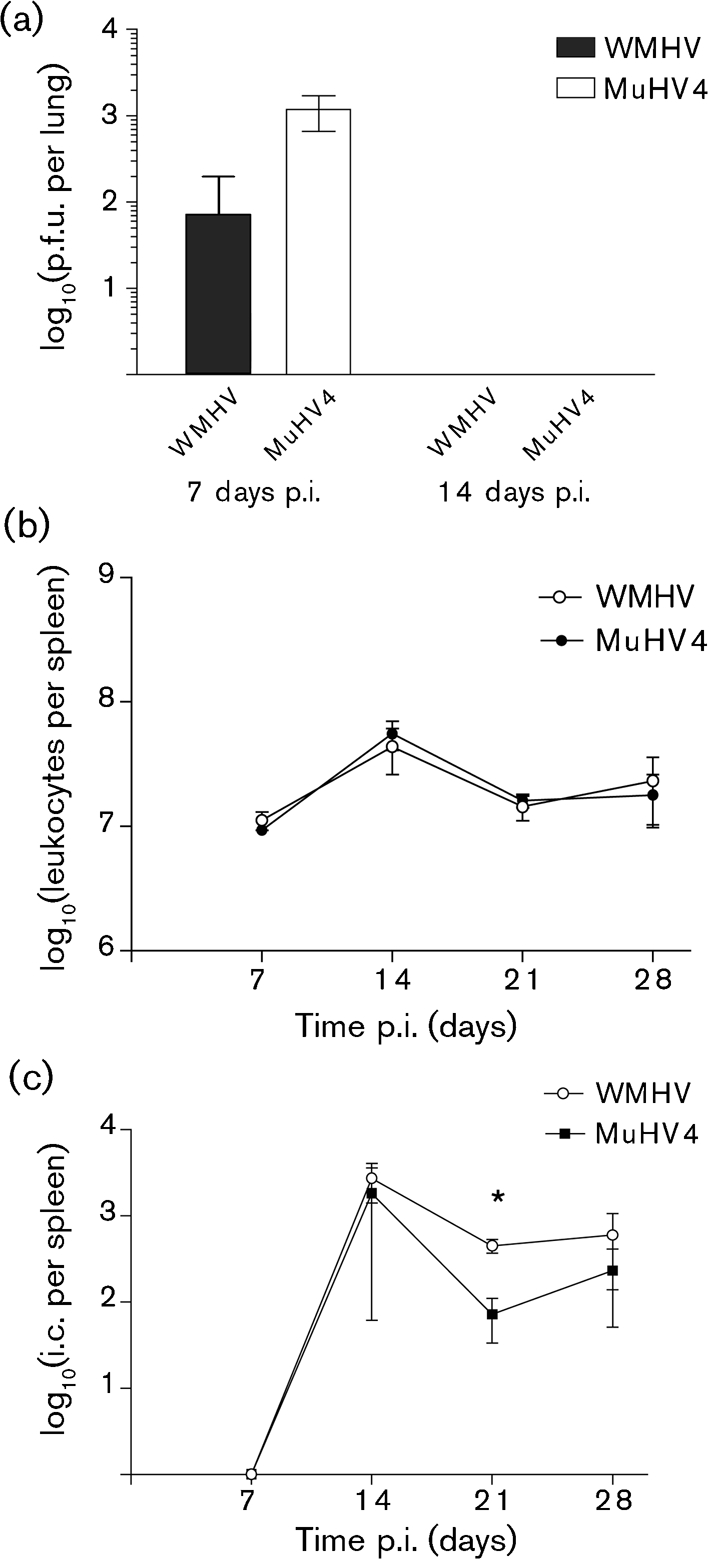
Virological analyses of WMHV infection of wood mice. Wood mice (three per time point) were infected intranasally with 4×10^5^ p.f.u. MuHV4 or WMHV. Results are shown as means±sd; the asterisk represents a statistically significant difference between species (*P*<0.05). (a) Infectious virus recovered from the lung at 7 and 14 days p.i. Titres were measured by plaque assay on NIH3T3 cells. (b) Mean leukocyte numbers per spleen. (c) Infective centre (i.c.) assay of the level of latency in splenocytes. Infectious virus titres in the samples were analysed in parallel and were subtracted from the number of total infectious centres.

Fig. 6 shows the features of infection of wood mice by WMHV in comparison with MuHV4. Infectious virus was detected in the lungs of wood mice infected with WMHV or MuHV4 at 7 days post-infection (p.i.), but not at 14 days p.i., and a significantly greater amount of infectious virus was recovered from MuHV4-infected wood mice at 7 days p.i. (Fig. 6a). The numbers of leukocytes per spleen isolated from infected wood mice were similar for WMHV and MuHV4 at all time points p.i. (Fig. 6b). There was an increase in the number of leukocytes at 14 days p.i. with both viruses, but this was marginal and transient, and infection with either virus did not induce significant splenomegaly. In WMHV- and MuHV4-infected wood mice, the number of latently infected cells per spleen increased dramatically from 7 days p.i., peaking at 14 days p.i. (Fig. 6c); the mean number of latently infected cells then declined approximately 6-fold by 21 days p.i. in WMHV-infected animals and 25-fold in the MuHV4-infected mice, and was largely unchanged at 28 days p.i. in both infections. The difference observed at 21 days p.i. was statistically significant (*P*<0.05).

**Fig. 7.**
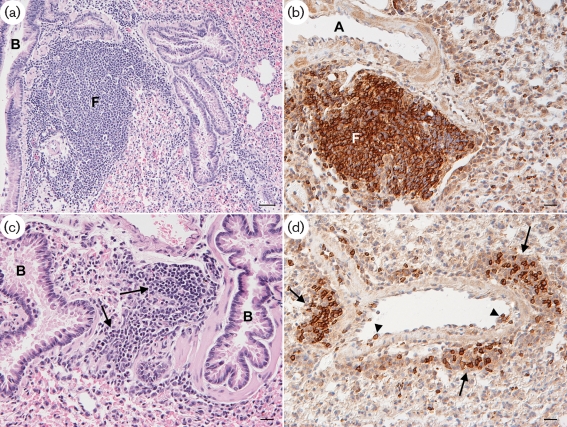
Cellular response to WMHV and MuHV4 infection in the lungs of wood mice at 14 days p.i. (a, b) Infection with MuHV4. (a) Intense peribronchial focal lymphocyte infiltration with evidence of lymphatic follicle formation (F). B, Bronchiole. (b) Focal perivascular B-cell infiltration with lymphatic follicle formation (F). A, Artery. (c, d) Infection with WMHV. (c) Moderate peribronchiolar focal lymphocyte infiltration (arrows). B, Bronchiole. (d) Artery with focal B-cell-dominated (CD45R-positive) perivascular lymphocyte infiltration (arrows). There is evidence of B-cell rolling and emigration (arrowheads). Sections were stained with haematoxylin and eosin (a, c), or for the B-cell marker CD45R, using the avidin–biotin–peroxidase complex method and Papanicolaou's haematoxylin counterstain (b, d). Bars, 50 μm (a); 20 μm (b–d).

Histological examination identified broadly similar changes in both WMHV- and MuHV4-infected wood mice that were similar to those reported in detail previously (Hughes *et al.*, 2010). On day 7 p.i., mild to moderate perivascular or peribronchial, B-cell-dominated lymphocyte infiltration with evidence of B-cell emigration from blood vessels was seen together with multifocal, predominantly perivascular macrophage and lymphocyte (i.e. granulomatous) infiltrates. There was a mild to moderate increase in the number of disseminated T and B cells in the interstitium. Viral antigen was scarce and was seen in occasional alveolar epithelial cells (type I and II pneumocytes) and in macrophages within the granulomatous infiltrates. The mediastinal lymph nodes and spleens of these animals contained primary and secondary follicles and unaltered T-cell zones. Rare viral antigen-positive macrophages were seen in the lymph nodes. At 14 days p.i., wood mice infected with MuHV4 displayed intense perivascular and peribronchial, B-cell-rich lymphocyte infiltration with evidence of lymphatic follicle formation (Fig. 7a, b). This has been described previously as iBALT (Hughes *et al.*, 2010). In contrast, in WMHV-infected animals, moderate multifocal perivascular and peribronchial B-cell infiltration and emigration was seen, but without distinct evidence of follicle formation (Fig. 7c, d). Granulomatous infiltrates were still observed in both groups; these contained macrophages exhibiting viral antigen. Large, well-delineated secondary follicles were observed in the spleens, and viral antigen-positive macrophages were detected in the red pulp. At 20 days p.i., both granulomatous infiltrates and perivascular and peribronchial lymphocyte infiltrations were still observed in the lungs. However, the follicle formation that was seen previously in MuHV4-infected wood mice had subsided. Spleens exhibited smaller secondary follicles than at day 14 p.i. Viral antigen-laden macrophages were seen in the spleen. In the lung, however, viral antigen expression was restricted to one individual macrophage in a granulomatous infiltrate in an MuHV4-infected animal. By day 28 p.i., the granulomatous infiltrates were few in number and small, but a mild to moderate perivascular and peribronchial lymphocyte infiltration remained. This persisted until 46 days p.i., to a mild degree. Thus, the changes observed were extremely similar except for the less intense B-cell infiltration and absence of iBALT in the lungs of WMHV-infected mice at day 14 p.i.

## DISCUSSION

This study demonstrated the abundance of herpesviruses in natural populations of wood mice in Cheshire. Two novel gammaherpesviruses, FVHV and WMHV, were isolated. Previous analyses have concluded that herpesvirus genomes of less than 95 % nucleotide sequence identity may represent different species (Ehlers *et al.*, 2007). Thus, the degree of divergence between WMHV and MuHV4, both overall (85 %) and within specific loci (e.g. *M2* and *ORF73*; Figs 2 and 3), is probably sufficient to warrant classification of WMHV as a new species. Under the current taxonomic scheme, in which murid herpesvirus species are named after the host family, this virus species would be *Murid herpesvirus 7*. Although an epidemiological study of free-living rodents in the UK was unable to distinguish between the two viruses (Blasdell *et al.*, 2003), a PCR-based study of mice trapped in Germany (Ehlers *et al.*, 2007) indicated that MuHV4 is present predominantly in yellow-necked field mice (*A. flavicollis*), whereas WMHV is present in wood mice (*A. sylvaticus*). However, MuHV4 was detected in some wood mice. Thus, it is possible that the two viruses normally infect different *Apodemus* species, but that there is some crossover. The biological characteristics of the two viruses in the wood mouse model exhibited significant similarities. However, the viruses did differ in their ability to grow in the lungs, in the development of iBALT and also perhaps in the efficiency of reactivation from splenic leukocytes. Interestingly, WMHV was isolated from trigeminal ganglia as well as spleens, suggesting that this virus may be neurotropic during a natural infection. This hypothesis warrants further investigation.

The analysis of the sequence of a large portion of the genome of the BRHV genome showed that the relationship of this virus to WMHV was sufficiently close (99 % identity) to warrant the consideration of WMHV and BRHV as strains of the same virus. Given that herpesviruses are thought generally to have evolved with their hosts (McGeoch *et al.*, 2006), this relationship was unanticipated, as the wood mouse and white-toothed shrew are classified in different mammalian orders, Rodentia (family Muridae) and Insectivora (family Soricidae), respectively. Thus, the claimed insectivore source of BRHV must be viewed as questionable. It is possible that the virus actually originated from a rodent, either by cross-infection in the wild or by laboratory contamination, as BRHV was isolated by passage in suckling mouse brains (Chastel *et al.*, 1994).

Other viruses related to MuHV4 have been characterized, but none so far has been shown to be sufficiently divergent from MuHV4 to form a new species. Viruses isolated from bank voles or yellow-necked field mice at the same time as MuHV4 (MHV-76, MHV-72, MHV-60 and MHV-78) are considered to be strains of MuHV4. MHV-76, although originally characterized as a novel alphaherpesvirus due to its cytopathic effect (CPE) *in vitro* (Ciampor *et al.*, 1981; Svobodova *et al.*, 1982) and then as a betaherpesvirus (Hamelin & Lussier, 1992), was conclusively demonstrated to be a gammaherpesvirus (Macrae *et al.*, 2001). MHV-76 proved to be equivalent to MuHV4 with a 9538 bp deletion at the left end of U, which probably arose during passage of the virus *in vivo* or *in vitro*. MHV-72 *ORF21* (encoding thymidine kinase) is identical in sequence to the corresponding MuHV4 gene (Raslova *et al.*, 2000), and *ORF51* (encoding gp150) differs by five nucleotide substitutions (Macakova *et al.*, 2003). Analysis of 12 other loci has shown that MHV-72 is more divergent from MuHV4 than MHV-76, and that *M1*, *M2* and *M3* are absent; none the less, MHV-72 and MuHV4 are highly related (Oda *et al.*, 2005). It seems likely that uncharacterized herpesviruses (MHV-60 and MHV-78) isolated at the same time as MuHV4 may also be strains of MuHV4 (Mistríková *et al.*, 2000; Nash *et al.*, 2001).

The WMHV genome is colinear with that of MuHV4, and the two viruses have the same predicted gene content (Figs 2 and 3). The reason for the generally higher degree of conservation of sequences near the left end of U is not known. Speculative explanations could centre on selective sweeps in this region of the genome or recombination between a WMHV-like virus and a virus more closely related to MuHV4. The non-coding sequences in this region, including the vtRNA-like transcripts, are generally highly conserved. However, there are differences in miRNAs 1, 2, 5, 6 and 9 derived from the primary transcripts of these vtRNAs (Fig. 4; Pfeffer *et al.*, 2004). The targets and exact functions of these miRNAs are not currently known (Pfeffer *et al.*, 2004), but these differences could have functional consequences and the comparative data could be informative. In addition to non-coding regions in this locus, the M1 and M4 proteins are highly conserved (Fig. 5). It has been proposed that the most likely function for the M4 protein is as a modulator of the innate immune system. *M4* is expressed *in vitro* with kinetics similar to immediate-early genes (Ebrahimi *et al.*, 2003), and *in vivo* it is expressed during productive infection but not during latency (Virgin *et al.*, 1999). M4 does not appear to have a role during the initial stages of infection *in vivo*, but is important during establishment of latency in the spleen (Evans *et al.*, 2006; Geere *et al.*, 2006). M1 has been shown to stimulate a V*β*4^+^ CD8^+^ T cell in a way reminiscent of a superantigen and, by doing this, to facilitate latent infection (Evans *et al.*, 2008).

The M3 protein, which is related to M1 and M4, is also well conserved, but somewhat less so than the M1 and M4 proteins, particularly towards the N terminus (Fig. 2). The secreted M3 protein is expressed strongly during lytic infection and probably to a lesser extent during latency (Simas *et al.*, 1999; Usherwood *et al.*, 2000; van Berkel *et al.*, 1999; Virgin *et al.*, 1999). *In vitro*, the M3 protein selectively binds chemokines associated with the antiviral inflammatory response (Parry *et al.*, 2000; van Berkel *et al.*, 2000). In the laboratory mouse, M3 was found to have a role in enhancing the amplification of latently infected B cells by affecting the CD8^+^ T-cell response (Bridgeman *et al.*, 2001), although this function was not seen in an independent study (van Berkel *et al.*, 2002). In the wood mouse model, M3 has a critical role in the amplification of latently infected B cells in the lung and the formation of iBALT containing these cells (Hughes *et al.*, 2010). Differences in M3 may therefore account for the lack of iBALT in WMHV-infected wood mice.

The M2 protein is the most divergent of the four proteins encoded by the left end of the genome and is associated with latency (Husain *et al.*, 1999). Numerous reports largely agree that *M2* is dispensable for long-term persistence, although MuHV4 recombinants lacking a functional *M2* gene are less efficient in the establishment of latency following intranasal infection of mice (Clambey *et al.*, 2002; Jacoby *et al.*, 2002; Macrae *et al.*, 2003; Simas *et al.*, 2004). It has also been postulated that *M2* is required for efficient colonization of follicle B cells and the development of these cells into memory B cells, a cell type exploited by MuHV4 for long-term latency (Simas *et al.*, 2004). Given the relationship, and possible overlap, between the hosts of WMHV and MuHV4, the divergence of the *M2* gene in a region of low overall variation might reflect strong immune selection. Indeed, it has been shown that an H2-Kd-restricted CD8^+^ T-cell epitope present in M2 (Husain *et al.*, 1999) sets the latent load during persistent infection of laboratory mice (*M. musculus*) (Marques *et al.*, 2008). However, this epitope is not conserved between MuHV4 and WMHV (Fig. 5b), suggesting that it may not be functional in the *Apodemus* hosts. The generation of greater numbers of infective centres (a measure of latency) in the spleens of the WMHV-infected wood mice at 21 days p.i. (Fig. 6c) raises the possibility that M2 may have evolved in this virus to augment the expansion of latently infected cells during the acute phase of latency. Experiments to address this hypothesis could involve replacing MuHV4 *M2* with WMHV *M2* and testing the phenotype in wood mice. Furthermore, numerous PXXP motifs are found throughout MuHV4 M2 (labelled P1–P9; Fig. 5b), some of which have been shown to functionally bind SH3-domain-containing proteins, such as Vav1 (Madureira *et al.*, 2005; Rodrigues *et al.*, 2006). Of these, P3, P4 and P5 have not been conserved in WMHV or BRHV. Recent *in vivo* analysis showed that mutations of P3, P4 or P5 had no effect on the establishment of, or reactivation from, splenic latency (Herskowitz *et al.*, 2008). Taken together, these motifs are unlikely to be important for the signalling function of M2. In a similar vein, the tyrosine residues at positions 120 and 129 of M2, which have been proven to be functional (Herskowitz *et al.*, 2008; Pires de Miranda *et al.*, 2008), are conserved in both WMHV and BRHV, highlighting their importance for the M2 signalling function.

The second most divergent protein in WMHV and MuHV4 is the virion glycoprotein gp150, encoded by *ORF51*. It seems likely that, in addition to exhibiting extensive differences in amino acid sequence, these proteins will be *N*-glycosylated differently in the two viruses (Fig. 5c). gp150 is a major target for the host antibody response, so it is likely to be under strong selective pressure (Gillet *et al.*, 2007). However, it is not clear why this membrane glycoprotein is more variable than others encoded by the two viruses.

The most divergent protein in WMHV and MuHV4 is encoded by *ORF73* (Fig. 5d). *In vivo* analyses of an MuHV4 mutant have shown that ORF73 is essential for the establishment and maintenance of latency (Fowler *et al.*, 2003), and preliminary characterization of *ORF73* mRNAs suggests that their transcription is similar to that of Kaposi's sarcoma-associated herpesvirus (KSHV; human herpesvirus 8) *ORF73* encoding the protein LANA (Coleman *et al.*, 2005). In a similar way to KSHV LANA, the MuHV4 ORF73 protein interacts with cellular bromodomain-containing BET proteins leading to activation of the promoters of G_1_/S cyclins (Ottinger *et al.*, 2009). The reason for the sequence variability in ORF73 is not clear. However, Epstein–Barr virus (EBV; human herpesvirus 4) EBNA1 (the functional analogue of rhadinovirus ORF73 proteins) shows considerable variability among strains (Wrightham *et al.*, 1995), and this has consequences for EBV-associated disease (Mai *et al.*, 2007; Wang *et al.*, 2003), the function of EBNA1 as a transcriptional transactivator (Do *et al.*, 2008) and the CD8^+^ T-cell response (Bell *et al.*, 2008).

In summary, WMHV is a novel MuHV4-like virus whose study will give further insight into gammaherpesvirus biology, especially in comparative terms alongside MuHV4.

## METHODS

### Cheshire herpesviruses

#### Isolation and growth.

Eight wood mice (WM1–WM8), a bank vole (BV1), a field vole (*Microtus agrestis*; FV1) and six house mice (HM1–HM6) were captured in Cheshire during 2002. The animals were killed by cervical dislocation and trigeminal ganglia, lungs and spleens were removed for virus reactivation. Virus was reactivated from trigeminal ganglia by explant culture as described previously (Efstathiou *et al.*, 1986). Virus was reactivated from the spleen using an infectious centre assay (Sunil-Chandra *et al.*, 1992). Lung tissue was homogenized and virus recovered as described previously (Stewart *et al.*, 1998). Mouse NIH3T3 cells (Todaro & Green, 1963) were used for all virus isolation experiments. Supernatants were examined as negatively stained preparations by TEM.

#### Preliminary sequence analysis.

Samples were tested for the presence of herpesvirus DPOL sequences by PCR. Whole-cell DNA from NIH3T3 cells was purified at 18–24 h p.i. using a QIAamp DNA Mini kit (Qiagen). PCR was carried out using the degenerate, deoxyinosine-substituted primers 5′-TGTAACTCGGTGTAYGGITTYACIGGIGT-3′ and 5′-CACAGAGTCCGTRTCICCRTAIAT-3′ (Ehlers *et al.*, 1999). PCR products were inserted into pCR2.1-TOPO (Invitrogen Life Technologies) and inserts were sequenced from three individual clones per product by Lark Technologies. Amino acid sequences deduced from the sequences of the PCR products were compared with known herpesvirus DPOL sequences using blast (Altschul *et al.*, 1997).

To amplify protein-coding DNA from genes *M1*, *M2* and *M3*, the samples were subjected to PCR using primers M1-f/M1-r (5′-TCATTGAGCAGCGGCGAC-3′ and 5′-GTATTCAGGCTTAGGACTG-3′; fragment size 1292 bp), M2-f/M2-r (5′-ATGGCCCCAACACCCCCAC-3′ and 5′-ACTCCTCGCCCCACTCCAC-3′; 577 bp) and M3-f/M3-r (5′-CTCTGGGAGAGCGTCAG-3′ and 5′-GTTACTGAGTATCAATGATCC-3′; 1251 bp), respectively. PCR products were sequenced as described above. The sequences obtained, minus those of the primers, accounted for the entire protein-coding region of each gene except for a few codons at one or both ends.

#### Genome sequence analysis.

Virus isolated from WM8 spleen was plaque purified three times from infected NIH3T3 cells overlaid with agarose, and a master stock of cell-associated virus was prepared and titrated. For the purposes of the present study, this virus was designated wood mouse herpesvirus (WMHV). WMHV was found to be primarily cell-associated in culture. To prepare virions for DNA extraction, WMHV was grown in a mouse cell line (*αβ*SV1) deficient in the response to alpha/beta interferon (IFN-*α*/*β*). This line was derived by first generating mouse embryonic fibroblasts (Todaro & Green, 1963) from IFN-*α*/*β* receptor knockout mice (Muller *et al.*, 1994). These cells were then transformed by transfection with a plasmid expressing simian virus 40 T antigen (from plasmid pVU0; Kalderon *et al.*, 1982) to generate an immortal cell line. The resulting cell line was found in preliminary experiments to release a much higher level of cell-free virus.

Twenty 150 cm^2^ tissue culture flasks of subconfluent *αβ*SV1 cells were infected with WMHV at an m.o.i. of 0.01 for 7 days. Virus DNA was then purified as described previously (Baldick *et al.*, 1997) and its integrity confirmed by agarose gel electrophoresis.

The DNA was sequenced at the Wellcome Trust Sanger Institute by a standard random shotgun approach to a mean coverage of 12 reads per nucleotide. Tandem repeat regions in the genome were determined using the program mreps (Kolpakov *et al.*, 2003), and the genome ends were inferred by comparison with the MuHV4 sequence (GenBank accession no. U97553; Virgin *et al.*, 1997). The main computer programs used to analyse the sequence were as follows: for sequence annotation, Artemis (Rutherford *et al.*, 2000), act (Carver *et al.*, 2005) and Sequin (NCBI); for sequence alignment, clustal w (Thompson *et al.*, 1994) and mafft (Katoh & Toh, 2008); for DNA sequence analyses, gcg (Accelrys) and emboss (Rice *et al.*, 2000); for amino acid sequence analysis, gcg, ExPASy (Gasteiger *et al.*, 2003), PTrans (Taylor, 1986) and Philius (Reynolds *et al.*, 2008); and for similarity searches, blast and fasta and its relatives (Pearson & Lipman, 1988).

#### Biological characterization.

The growth properties of WMHV were compared with those of MuHV4 in laboratory-bred wood mice using procedures described by Sunil-Chandra *et al.* (1992). All animal work was performed under UK Home Office Project Licence number 40/2483 and Personal Licence number 60/6501.

Wood mice were obtained from an outbred colony established at the Faculty of Veterinary Science, University of Liverpool, UK (Bennett *et al.*, 1997; Feore *et al.*, 1997). This colony was obtained from Dr J. Clarke in 1995, and was derived from captive-bred colonies that had been maintained for several decades in the Department of Zoology, University of Oxford, UK, with only occasional introductions of new stock from the wild. Their general housing and maintenance has been described elsewhere (Clarke, 1998), and at Liverpool they are maintained under semi-barrier conditions. The Liverpool colony has suffered no clinical disease, and, although not specified pathogen free in the sense used for most laboratory rodents, all samples tested for the major infections of laboratory rodents have so far been negative. Of particular relevance to this study, no evidence of MuHV4 infection has been detected by serology and PCR analysis (Blasdell *et al.*, 2003). Both male and female wood mice of 5–8 weeks of age were used. They were infected intranasally with 4×10^5^ p.f.u. virus, and the lungs, spleens and bronchial lymph nodes were harvested at various times p.i. Lung tissue was homogenized, and the lysate was freeze–thawed three times and used in plaque assays. Leukocytes were purified from the spleens and counted, and virus reactivation was monitored using an infective centre assay. Tissues from infected wood mice were routinely processed for histopathological examination, including immunohistology.

### Brest herpesvirus

#### Isolation and growth.

The herpesvirus Brest/AN711 isolated from a white-toothed shrew (Chastel *et al.*, 1994) was grown and titrated on baby hamster kidney cells as described previously (Bridgeman *et al.*, 2001). For the purposes of the present study, this virus was named Brest herpesvirus (BRHV). To prepare viral DNA, confluent monolayers of cells in 175 cm^2^ flasks were infected at an m.o.i. of 0.01. When CPE was complete at approximately 4 days p.i., virions were purified from the medium by Ficoll gradient ultracentrifugation as described previously (Lopes *et al.*, 2004). Banded virus was diluted with PBS to a total volume of 30 ml and pelleted at 30 000 ***g*** for 90 min. The pelleted virus was resuspended in Tris/EDTA (TE) buffer containing 0.5 % (w/v) SDS and 50 μg proteinase K ml^−1^. The mixture was incubated overnight at 37 °C and extracted with phenol, and the DNA was precipitated in ethanol and dissolved in a small volume of TE buffer, as described above.

#### Preliminary sequence analysis.

Initial cloning involved the generation of a small library of bacteriophage M13 recombinants containing BRHV *Alu*I fragments, using standard methods. The inserts in three recombinants were sequenced and found by a blast similarity search to be most closely related to the MuHV4 genome. Respectively, the insert sizes were 148, 145 and 156 bp and exhibited 89.2, 96.6 and 90.4 % nucleotide sequence identity to ORF18, ORF31 and ORF60.

#### Partial genome sequence analysis.

A cosmid library was generated from BRHV DNA as described previously (Cunningham & Davison, 1993). Three overlapping cosmid clones constituting approximately 70 kb of the genome were sequenced, the first by a standard random shotgun approach, and the other two by iterative primer walking on both strands, based initially on data generated from the first cosmid or arising from the preliminary sequence analysis described above. The computer programs used for analysis are listed above.

## Supplementary Material

[Supplementary Table]

## Figures and Tables

**Table 1.** Isolation of herpesviruses from free-living rodents in Cheshire

Animal*	**Tissues harvested†**	**Tissues yielding CPE†**	**Herpesvirus particles‡**
WM1	TG, S, L	TG, S	+
WM2	TG, S, L	S	+
WM3	TG, S, L	–	–
WM4	TG, S, L	–	–
WM5	TG, S, L	–	–
WM6	TG, S, L	–	–
WM7	TG, S, L	TG	+
WM8	TG, S, L	TG, S	+
BV1	TG, S, L	–	–
FV1	S, L	S	+
HM1	TG, S, L	–	–
HM2	TG, S, L	–	–
HM3	TG, S, L	–	–
HM4	TG, S, L	S	+
HM5	TG, S, L	–	–
HM6	TG, S, L	–	–

*WM, Wood mouse; BV, bank vole; FV, field vole; HM, house mouse; followed by a number for each animal. †TG, Trigeminal ganglia; S, spleen; L, lungs; –, no cytopathic effect (CPE). ‡+, Particles observed by TEM; –, particles not observed by TEM, on negatively stained preparations.

## References

[r1] Altschul, S. F., Madden, T. L., Schaffer, A. A., Zhang, J., Zhang, Z., Miller, W. & Lipman, D. J. (1997). Gapped blast and psi-blast: a new generation of protein database search programs. Nucleic Acids Res 25, 3389–3402. 9254694 10.1093/nar/25.17.3389 PMC146917

[r2] Baldick, C. J., Jr, Marchini, A., Patterson, C. E. & Shenk, T. (1997). Human cytomegalovirus tegument protein pp71 (ppUL82) enhances the infectivity of viral DNA and accelerates the infectious cycle. J Virol 71, 4400–4408. 9151830 10.1128/jvi.71.6.4400-4408.1997 PMC191658

[r3] Bell, M. J., Brennan, R., Miles, J. J., Moss, D. J., Burrows, J. M. & Burrows, S. R. (2008). Widespread sequence variation in Epstein–Barr virus nuclear antigen 1 influences the antiviral T cell response. J Infect Dis 197, 1594–1597. 18419576 10.1086/587848

[r4] Bennett, M., Crouch, A. J., Begon, M., Duffy, B., Feore, S., Gaskell, R. M., Kelly, D. F., McCracken, C. M., Vicary, L. & Baxby, D. (1997). Cowpox in British voles and mice. J Comp Pathol 116, 35–44. 9076598 10.1016/s0021-9975(97)80041-2

[r5] Blasdell, K., McCracken, C., Morris, A., Nash, A. A., Begon, M., Bennett, M. & Stewart, J. P. (2003). The wood mouse is a natural host for *Murid herpesvirus 4*. J Gen Virol 84, 111–113. 12533706 10.1099/vir.0.18731-0

[r6] Blaskovic, D., Stancekova, M., Svobodova, J. & Mistrikova, J. (1980). Isolation of five strains of herpesviruses from two species of free living small rodents. Acta Virol 24, 468 6111210

[r7] Bowden, R. J., Simas, J. P., Davis, A. J. & Efstathiou, S. (1997). Murine gammaherpesvirus 68 encodes tRNA-like sequences which are expressed during latency. J Gen Virol 78, 1675–1687. 9225045 10.1099/0022-1317-78-7-1675

[r8] Bridgeman, A., Stevenson, P. G., Simas, J. P. & Efstathiou, S. (2001). A secreted chemokine binding protein encoded by murine gammaherpesvirus-68 is necessary for the establishment of a normal latent load. J Exp Med 194, 301–312. 11489949 10.1084/jem.194.3.301 PMC2193474

[r9] Carver, T. J., Rutherford, K. M., Berriman, M., Rajandream, M. A., Barrell, B. G. & Parkhill, J. (2005). act: the Artemis Comparison Tool. Bioinformatics 21, 3422–3423. 15976072 10.1093/bioinformatics/bti553

[r10] Chastel, C., Beaucournu, J. P., Chastel, O., Legrand, M. C. & Le Goff, F. (1994). A herpesvirus from an European shrew (*Crocidura russula*). Acta Virol 38, 309 7726008

[r11] Ciampor, F., Stancekova, M. & Blaskovic, D. (1981). Electron microscopy of rabbit embryo fibroblasts infected with herpesvirus isolates from *Clethrionomys glareolus* and *Apodemus flavicollis*. Acta Virol 25, 101–107. 6113749

[r12] Clambey, E. T., Virgin, H. W., IV & Speck, S. H. (2002). Characterization of a spontaneous 9.5-kilobase-deletion mutant of murine gammaherpesvirus 68 reveals tissue-specific genetic requirements for latency. J Virol 76, 6532–6544. 12050366 10.1128/JVI.76.13.6532-6544.2002 PMC136253

[r13] Clarke, J. R. (1998). Voles. In *UFAW Handbook on the Care and Management of Laboratory Animals*. Edited by T. B. Poole. Oxford: Blackwell Scientific.

[r14] Coleman, H. M., Efstathiou, S. & Stevenson, P. G. (2005). Transcription of the murine gammaherpesvirus 68 ORF73 from promoters in the viral terminal repeats. J Gen Virol 86, 561–574. 15722515 10.1099/vir.0.80565-0

[r15] Cunningham, C. & Davison, A. J. (1993). A cosmid-based system for constructing mutants of herpes simplex virus type 1. Virology 197, 116–124. 8212547 10.1006/viro.1993.1572

[r16] Davison, A. J., Eberle, R., Ehlers, B., Hayward, G. S., McGeoch, D. J., Minson, A. C., Pellett, P. E., Roizman, B., Studdert, M. J. & Thiry, E. (2009). The order *Herpesvirales*. Arch Virol 154, 171–177. 19066710 10.1007/s00705-008-0278-4 PMC3552636

[r17] Do, N. V., Ingemar, E., Phi, P. T., Jenny, A., Chinh, T. T., Zeng, Y. & Hu, L. (2008). A major EBNA1 variant from Asian EBV isolates shows enhanced transcriptional activity compared to prototype B95.8. Virus Res 132, 15–24. 18096263 10.1016/j.virusres.2007.10.020

[r18] Ebrahimi, B., Dutia, B. M., Roberts, K. L., Garcia-Ramirez, J. J., Dickinson, P., Stewart, J. P., Ghazal, P., Roy, D. J. & Nash, A. A. (2003). Transcriptome profile of murine gammaherpesvirus-68 lytic infection. J Gen Virol 84, 99–109. 12533705 10.1099/vir.0.18639-0

[r19] Efstathiou, S., Minson, A. C., Field, H. J., Anderson, J. R. & Wildy, P. (1986). Detection of herpes simplex virus-specific DNA sequences in latently infected mice and in humans. J Virol 57, 446–455. 3003377 10.1128/jvi.57.2.446-455.1986 PMC252756

[r20] Efstathiou, S., Ho, Y. M., Hall, S., Styles, C. J., Scott, S. D. & Gompels, U. A. (1990). Murine herpesvirus 68 is genetically related to the gammaherpesviruses Epstein–Barr virus and herpesvirus saimiri. J Gen Virol 71, 1365–1372. 2161903 10.1099/0022-1317-71-6-1365

[r21] Ehlers, B., Borchers, K., Grund, C., Frolich, K., Ludwig, H. & Buhk, H. J. (1999). Detection of new DNA polymerase genes of known and potentially novel herpesviruses by PCR with degenerate and deoxyinosine-substituted primers. Virus Genes 18, 211–220. 10456789 10.1023/a:1008064118057

[r22] Ehlers, B., Kuchler, J., Yasmum, N., Dural, G., Voigt, S., Schmidt-Chanasit, J., Jakel, T., Matuschka, F. R., Richter, D. & other authors (2007). Identification of novel rodent herpesviruses, including the first gammaherpesvirus of *Mus musculus*. J Virol 81, 8091–8100. 17507487 10.1128/JVI.00255-07 PMC1951306

[r23] Evans, A. G., Moorman, N. J., Willer, D. O. & Speck, S. H. (2006). The M4 gene of *γ*HV68 encodes a secreted glycoprotein and is required for the efficient establishment of splenic latency. Virology 344, 520–531. 16185740 10.1016/j.virol.2005.08.020

[r24] Evans, A. G., Moser, J. M., Krug, L. T., Pozharskaya, V., Mora, A. L. & Speck, S. H. (2008). A gammaherpesvirus-secreted activator of V*β*4^+^ CD8^+^ T cells regulates chronic infection and immunopathology. J Exp Med 205, 669–684. 18332178 10.1084/jem.20071135 PMC2275388

[r25] Feore, S. M., Bennett, M., Chantrey, J., Jones, T., Baxby, D. & Begon, M. (1997). The effect of cowpox virus infection on fecundity in bank voles and wood mice. Proc Biol Sci 264, 1457–1461. 9364786 10.1098/rspb.1997.0202 PMC1688698

[r26] Fowler, P., Marques, S., Simas, J. P. & Efstathiou, S. (2003). ORF73 of murine herpesvirus-68 is critical for the establishment and maintenance of latency. J Gen Virol 84, 3405–3416. 14645921 10.1099/vir.0.19594-0

[r27] Gasteiger, E., Gattiker, A., Hoogland, C., Ivanyi, I., Appel, R. D. & Bairoch, A. (2003). ExPASy: the proteomics server for in-depth protein knowledge and analysis. Nucleic Acids Res 31, 3784–3788. 12824418 10.1093/nar/gkg563 PMC168970

[r28] Geere, H. M., Ligertwood, Y., Templeton, K. M., Bennet, I., Gangadharan, B., Rhind, S. M., Nash, A. A. & Dutia, B. M. (2006). The M4 gene of murine gammaherpesvirus 68 modulates latent infection. J Gen Virol 87, 803–807. 16528028 10.1099/vir.0.81577-0

[r29] Gillet, L., May, J. S., Colaco, S. & Stevenson, P. G. (2007). The murine gammaherpesvirus-68 gp150 acts as an immunogenic decoy to limit virion neutralization. PLoS One 2, e705 17684552 10.1371/journal.pone.0000705 PMC1931612

[r30] Hamelin, C. & Lussier, G. (1992). Characterization of the DNA of rodent herpesviruses by restriction endonuclease analysis and hybridization. Lab Anim Sci 42, 142–145. 1318445

[r31] Herskowitz, J. H., Siegel, A. M., Jacoby, M. A. & Speck, S. H. (2008). Systematic mutagenesis of the murine gammaherpesvirus 68 M2 protein identifies domains important for chronic infection. J Virol 82, 3295–3310. 18234799 10.1128/JVI.02234-07 PMC2268483

[r32] Hughes, D. J., Kipar, A., Sample, J. T. & Stewart, J. P. (2010). Pathogenesis of a model gammaherpesvirus in a natural host. J Virol (Feb 3; Epub ahead of print) doi:10.1128/JVI.02085-09.. 10.1128/JVI.02085-09 PMC2849519 20130062

[r33] Husain, S. M., Usherwood, E. J., Dyson, H., Coleclough, C., Coppola, M. A., Woodland, D. L., Blackman, M. A., Stewart, J. P. & Sample, J. T. (1999). Murine gammaherpesvirus M2 gene is latency-associated and its protein a target for CD8^+^ T lymphocytes. Proc Natl Acad Sci U S A 96, 7508–7513. 10377445 10.1073/pnas.96.13.7508 PMC22116

[r34] Jacoby, M. A., Virgin, H. W., IV & Speck, S. H. (2002). Disruption of the M2 gene of murine gammaherpesvirus 68 alters splenic latency following intranasal, but not intraperitoneal, inoculation. J Virol 76, 1790–1801. 11799175 10.1128/JVI.76.4.1790-1801.2002 PMC135904

[r35] Kalderon, D., Oostra, B. A., Ely, B. K. & Smith, A. E. (1982). Deletion loop mutagenesis: a novel method for the construction of point mutations using deletion mutants. Nucleic Acids Res 10, 5161–5171. 6292832 10.1093/nar/10.17.5161 PMC320862

[r36] Katoh, K. & Toh, H. (2008). Recent developments in the mafft multiple sequence alignment program. Brief Bioinform 9, 286–298. 18372315 10.1093/bib/bbn013

[r37] Kolpakov, R., Bana, G. & Kucherov, G. (2003). mreps: efficient and flexible detection of tandem repeats in DNA. Nucleic Acids Res 31, 3672–3678. 12824391 10.1093/nar/gkg617 PMC169196

[r38] Lopes, F. B., Colaco, S., May, J. S. & Stevenson, P. G. (2004). Characterization of murine gammaherpesvirus 68 glycoprotein B. J Virol 78, 13370–13375. 15542690 10.1128/JVI.78.23.13370-13375.2004 PMC525018

[r39] Macakova, K., Matis, J., Rezuchova, I., Kudela, O., Raslova, H. & Kudelova, M. (2003). Murine gammaherpesvirus (MHV) M7 gene encoding glycoprotein 150 (gp150): difference in the sequence between 72 and 68 strains. Virus Genes 26, 89–95. 12683351 10.1023/a:1022390407991

[r40] Macrae, A. I., Dutia, B. M., Milligan, S., Brownstein, D. G., Allen, D. J., Mistrikova, J., Davison, A. J., Nash, A. A. & Stewart, J. P. (2001). Analysis of a novel strain of murine gammaherpesvirus reveals a genomic locus important for acute pathogenesis. J Virol 75, 5315–5327. 11333912 10.1128/JVI.75.11.5315-5327.2001 PMC114936

[r41] Macrae, A. I., Usherwood, E. J., Husain, S. M., Flano, E., Kim, I. J., Woodland, D. L., Nash, A. A., Blackman, M. A., Sample, J. T. & Stewart, J. P. (2003). Murid herpesvirus 4 strain 68 M2 protein is a B-cell-associated antigen important for latency but not lymphocytosis. J Virol 77, 9700–9709. 12915582 10.1128/JVI.77.17.9700-9709.2003 PMC187398

[r42] Madureira, P. A., Matos, P., Soeiro, I., Dixon, L. K., Simas, J. P. & Lam, E. W. (2005). Murine gamma-herpesvirus 68 latency protein M2 binds to Vav signaling proteins and inhibits B-cell receptor-induced cell cycle arrest and apoptosis in WEHI-231 B cells. J Biol Chem 280, 37310–37318. 16150693 10.1074/jbc.M507478200

[r43] Mai, S. J., Ooka, T., Li, D. J., Zeng, M. S., Jiang, R. C., Yu, X. J., Zhang, R. H., Chen, S. P. & Zeng, Y. X. (2007). Functional advantage of NPC-related V-val subtype of Epstein–Barr virus nuclear antigen 1 compared with prototype in epithelial cell line. Oncol Rep 17, 141–146. 17143491

[r44] Marques, S., Alenquer, M., Stevenson, P. G. & Simas, J. P. (2008). A single CD8^+^ T cell epitope sets the long-term latent load of a murid herpesvirus. PLoS Pathog 4, e1000177 18846211 10.1371/journal.ppat.1000177 PMC2556087

[r45] McGeoch, D. J., Gatherer, D. & Dolan, A. (2005). On phylogenetic relationships among major lineages of the *Gammaherpesvirinae*. J Gen Virol 86, 307–316. 15659749 10.1099/vir.0.80588-0

[r46] McGeoch, D. J., Rixon, F. J. & Davison, A. J. (2006). Topics in herpesvirus genomics and evolution. Virus Res 117, 90–104. 16490275 10.1016/j.virusres.2006.01.002

[r47] Mistríková, J., Raslova, H., Mrmusova, M. & Kudelova, M. (2000). A murine gammaherpesvirus. Acta Virol 44, 211–226. 11155368

[r48] Muller, U., Steinhoff, U., Reis, L. F., Hemmi, S., Pavlovic, J., Zinkernagel, R. M. & Aguet, M. (1994). Functional role of type I and type II interferons in antiviral defense. Science 264, 1918–1921. 8009221 10.1126/science.8009221

[r49] Nash, A. A., Dutia, B. M., Stewart, J. P. & Davison, A. J. (2001). Natural history of murine gamma-herpesvirus infection. Philos Trans R Soc Lond B Biol Sci 356, 569–579. 11313012 10.1098/rstb.2000.0779 PMC1088445

[r50] Oda, W., Mistrikova, J., Stancekova, M., Dutia, B. M., Nash, A. A., Takahata, H., Jin, Z., Oka, T. & Hayashi, K. (2005). Analysis of genomic homology of murine gammaherpesvirus (MHV)-72 to MHV-68 and impact of MHV-72 on the survival and tumorigenesis in the MHV-72-infected CB17^scid/scid^ and CB17^+/+^ mice. Pathol Int 55, 558–568. 16143031 10.1111/j.1440-1827.2005.01869.x

[r51] Ottinger, M., Pliquet, D., Christalla, T., Frank, R., Stewart, J. P. & Schulz, T. F. (2009). The interaction of the gammaherpesvirus 68 orf73 protein with cellular BET proteins affects the activation of cell cycle promoters. J Virol 83, 4423–4434. 19244327 10.1128/JVI.02274-08 PMC2668493

[r52] Parry, C. M., Simas, J. P., Smith, V. P., Stewart, C. A., Minson, A. C., Efstathiou, S. & Alcami, A. (2000). A broad spectrum secreted chemokine binding protein encoded by a herpesvirus. J Exp Med 191, 573–578. 10662803 10.1084/jem.191.3.573 PMC2195820

[r53] Pearson, W. R. & Lipman, D. J. (1988). Improved tools for biological sequence comparison. Proc Natl Acad Sci U S A 85, 2444–2448. 3162770 10.1073/pnas.85.8.2444 PMC280013

[r54] Pfeffer, S., Zavolan, M., Grasser, F. A., Chien, M., Russo, J. J., Ju, J., John, B., Enright, A. J., Marks, D. & other authors (2004). Identification of virus-encoded microRNAs. Science 304, 734–736. 15118162 10.1126/science.1096781

[r55] Pires de Miranda, M., Alenquer, M., Marques, S., Rodrigues, L., Lopes, F., Bustelo, X. R. & Simas, J. P. (2008). The gammaherpesvirus m2 protein manipulates the Fyn/Vav pathway through a multidocking mechanism of assembly. PLoS One 3, e1654 18301737 10.1371/journal.pone.0001654 PMC2244710

[r56] Raslova, H., Matis, J., Rezuchova, I., Macakova, K., Berebbi, M. & Kudelova, M. (2000). The bystander effect mediated by the new murine gammaherpesvirus 72–thymidine kinase/5′-fluoro-2′-deoxyuridine (MHV72–TK/5-FUdR) system in vitro. Antivir Chem Chemother 11, 273–282. 10950389 10.1177/095632020001100403

[r57] Reynolds, S. M., Kall, L., Riffle, M. E., Bilmes, J. A. & Noble, W. S. (2008). Transmembrane topology and signal peptide prediction using dynamic Bayesian networks. PLOS Comput Biol 4, e1000213 18989393 10.1371/journal.pcbi.1000213 PMC2570248

[r58] Rice, P., Longden, I. & Bleasby, A. (2000). emboss: the European Molecular Biology Open Software Suite. Trends Genet 16, 276–277. 10827456 10.1016/s0168-9525(00)02024-2

[r59] Rodrigues, L., Pires de Miranda, M., Caloca, M. J., Bustelo, X. R. & Simas, J. P. (2006). Activation of Vav by the gammaherpesvirus M2 protein contributes to the establishment of viral latency in B lymphocytes. J Virol 80, 6123–6135. 16731951 10.1128/JVI.02700-05 PMC1472561

[r60] Rutherford, K., Parkhill, J., Crook, J., Horsnell, T., Rice, P., Rajandream, M. A. & Barrell, B. (2000). Artemis: sequence visualization and annotation. Bioinformatics 16, 944–945. 11120685 10.1093/bioinformatics/16.10.944

[r61] Simas, J. P., Swann, D., Bowden, R. & Efstathiou, S. (1999). Analysis of murine gammaherpesvirus-68 transcription during lytic and latent infection. J Gen Virol 80, 75–82. 9934687 10.1099/0022-1317-80-1-75

[r62] Simas, J. P., Marques, S., Bridgeman, A., Efstathiou, S. & Adler, H. (2004). The M2 gene product of murine gammaherpesvirus 68 is required for efficient colonization of splenic follicles but is not necessary for expansion of latently infected germinal centre B cells. J Gen Virol 85, 2789–2797. 15448339 10.1099/vir.0.80138-0

[r63] Stewart, J. P., Usherwood, E. J., Ross, A., Dyson, H. & Nash, T. (1998). Lung epithelial cells are a major site of murine gammaherpesvirus persistence. J Exp Med 187, 1941–1951. 9625754 10.1084/jem.187.12.1941 PMC2212355

[r64] Sunil-Chandra, N. P., Efstathiou, S., Arno, J. & Nash, A. A. (1992). Virological and pathological features of mice infected with murine gamma-herpesvirus 68. J Gen Virol 73, 2347–2356. 1328491 10.1099/0022-1317-73-9-2347

[r65] Svobodova, J., Blaskovic, D. & Mistrikova, J. (1982). Growth characteristics of herpesviruses isolated from free living small rodents. Acta Virol 26, 256–263. 6127933

[r66] Taylor, P. (1986). A computer program for translating DNA sequences into protein. Nucleic Acids Res 14, 437–441. 3753779 10.1093/nar/14.1.437 PMC339428

[r67] Thompson, J. D., Higgins, D. G. & Gibson, T. J. (1994). clustal w: improving the sensitivity of progressive multiple sequence alignment through sequence weighting, positions-specific gap penalties and weight matrix choice. Nucleic Acids Res 22, 4673–4680. 7984417 10.1093/nar/22.22.4673 PMC308517

[r68] Todaro, G. J. & Green, H. (1963). Quantitative studies of the growth of mouse embryo cells in culture and their development into established lines. J Cell Biol 17, 299–313. 13985244 10.1083/jcb.17.2.299 PMC2106200

[r69] Usherwood, E. J., Roy, D. J., Ward, K., Surman, S. L., Dutia, B. M., Blackman, M. A., Stewart, J. P. & Woodland, D. L. (2000). Control of gammaherpesvirus latency by latent antigen-specific CD8^+^ T cells. J Exp Med 192, 943–952. 11015436 10.1084/jem.192.7.943 PMC2193320

[r70] van Berkel, V., Preiter, K., Virgin, H. W. & Speck, S. H. (1999). Identification and initial characterization of the murine gammaherpesvirus 68 gene M3, encoding an abundantly secreted protein. J Virol 73, 4524–4529. 10196360 10.1128/jvi.73.5.4524-4529.1999 PMC104349

[r71] van Berkel, V., Barrett, J., Tiffany, H. L., Fremont, D. H., Murphy, P. M., McFadden, G., Speck, S. H. & Virgin, H. W. (2000). Identification of a gammaherpesvirus selective chemokine binding protein that inhibits chemokine action. J Virol 74, 6741–6747. 10888612 10.1128/jvi.74.15.6741-6747.2000 PMC112190

[r72] van Berkel, V., Levine, B., Kapadia, S. B., Goldman, J. E., Speck, S. H. & Virgin, H. W., IV (2002). Critical role for a high-affinity chemokine-binding protein in gamma-herpesvirus-induced lethal meningitis. J Clin Invest 109, 905–914. 11927617 10.1172/JCI14358 PMC150927

[r73] Virgin, H. W., Latreille, P., Wamsley, P., Hallsworth, K., Weck, K. E., Dal Canto, A. J. & Speck, S. H. (1997). Complete sequence and genomic analysis of murine gammaherpesvirus 68. J Virol 71, 5894–5904. 9223479 10.1128/jvi.71.8.5894-5904.1997 PMC191845

[r74] Virgin, H. W., Presti, R. M., Li, X. Y., Liu, C. & Speck, S. H. (1999). Three distinct regions of the murine gammaherpesvirus 68 genome are transcriptionally active in latently infected mice. J Virol 73, 2321–2332. 9971815 10.1128/jvi.73.3.2321-2332.1999 PMC104477

[r75] Wang, J. T., Sheeng, T. S., Su, I. J., Chen, J. Y. & Chen, M. R. (2003). EBNA-1 sequence variations reflect active EBV replication and disease status or quiescent latency in lymphocytes. J Med Virol 69, 417–425. 12526054 10.1002/jmv.10305

[r76] Wrightham, M. N., Stewart, J. P., Janjua, N. J., Pepper, S. D., Sample, C., Rooney, C. M. & Arrand, J. R. (1995). Antigenic and sequence variation in the C-terminal unique domain of the Epstein–Barr virus nuclear antigen EBNA-1. Virology 208, 521–530. 7538250 10.1006/viro.1995.1183

