# Supplementary material for: Characterization of a novel wood mouse virus related to murid herpesvirus 4
Source: J Gen Virol. 2010 Apr;91(Pt 4):867–79. doi: 10.1099/vir.0.017327-0 (PMC2888160; doi:10.1099/vir.0.017327-0)
Supplement: [Supplementary Table] [file supp_91_4_867__index.html]

 Characterization of a novel wood mouse virus related to murid herpesvirus 4 -- Hughes et al. 91 (4): 867 Data Supplement - Supplementary Table -- Journal of General Virology

### Characterization of a novel wood mouse virus related to murid herpesvirus 4, by D. J . Hughes, A. Kipar, S. G. Milligan, C. Cunningham, M. Sanders, M. A. Quail, M.-A. Rajandream, S. Efstathiou, R. J. Bowden, C. Chastel, M. Bennett, J. T. Sample, B. Barrell, A. J. Davison and J. P. Stewart

*Journal of General Virology* vol. **91**, part 4, pp. 867 –879

  

**Supplementary Table S1.** Information used to annotate the WMHV and MuHV4 genome sequences [PDF] (65 KB)

  
  
