# Supplementary material for: Characterization of a novel wood mouse virus related to murid herpesvirus 4
Source: J Gen Virol. 2010 Apr;91(Pt 4):867–79. doi: 10.1099/vir.0.017327-0 (PMC2888160; doi:10.1099/vir.0.017327-0)
Supplement: [Supplementary Table] [file supp_91_4_867__1.pdf]

Supplementary Table S1. Information used to annotate the WMHV and MuHV4 genome sequences

[purple box] Encoded by a core gene (i.e. inherited from an ancestor of alpha-, beta- and gammaherpesviruses);

[blue box] Encoded by a betagamma gene (i.e. inherited from an ancestor of beta- and gammaherpesviruses);

[orange box] Encoded by a gamma gene (i.e. inherited from an ancestor of gammaherpesviruses);

[white box] Encoded by a gene specific to a subset of gammaherpesviruses.

Gene	Protein name*	Family	Description	EC no.	Activity
M1	Protein M1	M1	Contains a signal peptide; secreted protein		Unknown
M2	Protein M2		Interacts with Vav oncoprotein; involved in B-cell proliferation and differentiation		Immune regulation; latency
M3	Chemokine-binding protein M3	M1	Contains a signal peptide; secreted protein		Immune regulation
M4	Chemokine-binding protein M4	M1	Contains a signal peptide; secreted protein		Immune regulation
ORF4	Complement control protein	CCP	Type 1 envelope glycoprotein; contains four SCR domains		Immune regulation
ORF6	Single-stranded DNA-binding protein		Contains a zinc finger		DNA replication; possibly gene regulation
ORF7	DNA packaging terminase subunit 2				DNA encapsidation
ORF8	Envelope glycoprotein B		Type 1 membrane protein; possible membrane fusogen; binds cell-surface heparan sulphate		Cell entry; cell-to-cell spread
ORF9	DNA polymerase catalytic subunit			2.7.7.7	DNA replication
ORF10	Protein G10	DURP			Unknown
ORF11	Virion protein G11	DURP			Unknown
ORF12	E3 ubiquitin ligase MIR1	MIR	Type 3 membrane protein; two transmembrane domains; contains a PHD finger; downregulates MHC I		Immune regulation
13M	Protein 13M		Hypothetical protein encoded by 5′-region of ORF12 transcript		Unknown
ORF17.5	Capsid scaffold protein		Clipped near C terminus		Capsid morphogenesis

Hughes, D. J ., Kipar, A., Milligan, S. G., Cunningham, C., Sanders, M., Quail, M. A., Rajandream, M.-A., Efstathiou, S., Bowden, R. J., Chastel, C., Bennett, M., Sample, J. T., Barrell, B., Davison, A. J. & Stewart, J. P. (2010). Characterization of a novel wood mouse virus related to murid herpesvirus 4. *J Gen Virol* **91**, 867–879.

Gene	Protein name*	Family	Description	EC no.	Activity
ORF17	Capsid maturation protease		Serine protease (N-terminal region); minor scaffold protein (remainder of protein, clipped near C terminus)	3.4.21.97	Capsid morphogenesis
ORF18	Protein UL79		Required for expression of late genes		Gene regulation
ORF19	DNA packaging tegument protein UL25		Located on capsid near vertices; possibly stabilizes the capsid and retains the genome		DNA encapsidation
ORF20	Nuclear protein UL24				Unknown
ORF21	Thymidine kinase			2.7.1.21	Nucleotide metabolism
ORF22	Envelope glycoprotein H		Type 1 membrane protein; possible membrane fusogen; complexed with envelope glycoprotein L		Cell entry; cell-to-cell spread
ORF23	Protein UL88				Unknown
ORF24	Virion protein UL87				Unknown
ORF25	Major capsid protein		Six copies form hexons, five copies form pentons		Capsid morphogenesis
ORF26	Capsid triplex subunit 2		Complexed 2 : 1 with capsid triplex subunit 1 to connect capsid hexons and pentons		Capsid morphogenesis
ORF27	Envelope glycoprotein 48		Type 2 membrane protein		Cell-to-cell spread
ORF28	Envelope glycoprotein 150		Type 1 membrane protein		Immune regulation
ORF29	DNA packaging terminase subunit 1		Contains an ATPase domain		DNA encapsidation
ORF30	Protein UL91				Unknown
ORF31	Protein UL92				Unknown
ORF32	DNA packaging tegument protein UL17		Capsid-associated		DNA encapsidation; capsid transport
ORF33	Tegument protein UL16				Possibly virion morphogenesis
ORF34	Protein UL95				Unknown
ORF35	Tegument protein UL14				Virion morphogenesis
ORF36	Tegument serine/threonine protein kinase	PK		2.7.11.1	Protein phosphorylation
ORF37	Deoxyribonuclease				DNA processing
ORF38	Myristylated tegument protein		Envelope-associated		Virion morphogenesis
ORF39	Envelope glycoprotein M		Type 3 membrane protein; eight transmembrane domains; complexed with envelope glycoprotein N		Virion morphogenesis; membrane fusion
ORF40	Helicase–primase subunit				DNA replication
ORF42	Tegument protein UL7				Virion morphogenesis

Hughes, D. J ., Kipar, A., Milligan, S. G., Cunningham, C., Sanders, M., Quail, M. A., Rajandream, M.-A., Efstathiou, S., Bowden, R. J., Chastel, C., Bennett, M., Sample, J. T., Barrell, B., Davison, A. J. & Stewart, J. P. (2010). Characterization of a novel wood mouse virus related to murid herpesvirus 4. *J Gen Virol* **91**, 867–879.

Gene	Protein name*	Family	Description	EC no.	Activity
ORF43	Capsid portal protein		Dodecamer located at one capsid vertex in place of a penton		DNA encapsidation
ORF44	Helicase–primase helicase subunit				DNA replication
ORF45	Tegument protein G45				Unknown
ORF46	Uracil-DNA glycosylase			3.2.2.3	DNA repair
ORF47	Envelope glycoprotein L		Complexed with envelope glycoprotein H		Cell entry; cell-to-cell spread
ORF48	Tegument protein G48				Unknown
ORF49	Protein G49		Cooperates with protein Rta		Gene regulation
ORF50	Protein Rta				Gene regulation; latency
ORF51	Envelope glycoprotein 350		Type 1 membrane protein		Cell attachment
ORF52	Virion protein G52				Unknown
ORF53	Envelope glycoprotein N		Type 1 membrane protein; complexed with envelope glycoprotein M		Virion morphogenesis; membrane fusion
ORF54	Deoxyuridine triphosphatase	DURP		3.6.1.23	Nucleotide metabolism
ORF55	Tegument protein UL51				Virion morphogenesis
ORF56	Helicase–primase primase subunit				DNA replication
ORF57	Multifunctional expression regulator	MER	RNA-binding protein; shuttles between nucleus and cytoplasm; inhibits pre-mRNA splicing; exports virus mRNA from nucleus; exerts most effects post-transcriptionally		Gene regulation; RNA metabolism and transport
ORF58	Envelope protein UL43		Type 3 membrane protein; 11 transmembrane domains		Possibly membrane fusion
ORF59	DNA polymerase processivity subunit		dsDNA-binding protein		DNA replication
ORF60	Ribonucleotide reductase subunit 2			1.17.4.1	Nucleotide metabolism
ORF61	Ribonucleotide reductase subunit 1			1.17.4.1	Nucleotide metabolism
ORF62	Capsid triplex subunit 1		Complexed 1 : 2 with capsid triplex subunit 2 to connect capsid hexons and pentons		Capsid morphogenesis
ORF63	Tegument protein UL37		Complexed with large tegument protein		Virion morphogenesis
ORF64	Large tegument protein		Complexed with tegument protein UL37; ubiquitin-specific protease (N-terminal region)		Capsid transport

Hughes, D. J ., Kipar, A., Milligan, S. G., Cunningham, C., Sanders, M., Quail, M. A., Rajandream, M.-A., Efstathiou, S., Bowden, R. J., Chastel, C., Bennett, M., Sample, J. T., Barrell, B., Davison, A. J. & Stewart, J. P. (2010). Characterization of a novel wood mouse virus related to murid herpesvirus 4. *J Gen Virol* **91**, 867–879.

Gene	Protein name*	Family	Description	EC no.	Activity
ORF65	Small capsid protein		Located externally on capsid hexons		Capsid morphogenesis; possibly capsid transport
ORF66	Protein UL49				Unknown
ORF67	Nuclear egress type 2 Membrane protein		Interacts with nuclear egress lamina protein		Nuclear egress
ORF67A	DNA-packaging protein UL33		Interacts with DNA packaging terminase subunit 2		DNA encapsidation
ORF68	DNA-packaging protein UL32				DNA encapsidation; possibly capsid transport
ORF69	Nuclear egress lamina protein		Interacts with nuclear egress type 2 membrane protein		Nuclear egress
ORF72	Cyclin				Cell cycle regulation
M11	Apoptosis regulator M11	Bcl-2			Apoptosis
ORF73	Nuclear antigen LANA		Chromosome-tethering protein		Latency
ORF74	Membrane protein G74	GPCR	Type 3 membrane protein; seven transmembrane domains		Intracellular signalling
ORF75C	Tegument protein G75C	FGARAT			Unknown
ORF75B	Protein G75B	FGARAT			Unknown
ORF75A	Protein G75A	FGARAT			Unknown

*Protein names are a provisional standard for all herpesviruses.

Hughes, D. J ., Kipar, A., Milligan, S. G., Cunningham, C., Sanders, M., Quail, M. A., Rajandream, M.-A., Efstathiou, S., Bowden, R. J., Chastel, C., Bennett, M., Sample, J. T., Barrell, B., Davison, A. J. & Stewart, J. P. (2010). Characterization of a novel wood mouse virus related to murid herpesvirus 4. *J Gen Virol* **91**, 867–879.
